# The first cytogenetic description of *Euleptes europaea* (Gené, 1839) from Northern Sardinia reveals the highest diploid chromosome number among sphaerodactylid geckos (Sphaerodactylidae, Squamata)

**DOI:** 10.3897/CompCytogen.v7i2.4881

**Published:** 2013-06-10

**Authors:** Ekaterina Gornung, Fabio Mosconi, Flavia Annesi, Riccardo Castiglia

**Affiliations:** 1 Dipartimento di Biologia e Biotecnologie “Charles Darwin”, Università di Roma “La Sapienza”, Via Alfonso Borelli 50 – 00161 – Roma – Italia; 2 Cooperativa Myosotis c/o Museo Civico di Zoologia di Roma, Via Aldrovandi 18 – 00197 – Roma – Italia

**Keywords:** Sauria, Gekkota, karyotype, chromosomal evolution, telomeric repeats, XY male heterogamety

## Abstract

The karyotype of a sphaerodactylid gecko *Euleptes europaea* (Gené, 1839) was assembled for the first time in this species. It is made of 2n = 42 gradually decreasing in size chromosomes, the highest chromosome number so far acknowledged in the family Sphaerodactylidae. The second chromosome pair of the karyotype appears slightly heteromorphic in the male individual. Accordingly, FISH with a telomeric probe revealed an uneven distribution of telomeric repeats on the two homologues of this pair, which may be indicative of an XY sex-determination system in the species, to be further investigated.

## Introduction

The Italian Gekkotan fauna includes four species: two gekkonid species – *Mediodactylus kotschyi* (Steindachner, 1870) and *Hemidactylus turcicus* (Linnaeus, 1758), a phyllodactylid gecko *Tarentola mauritanica* (Linnaeus, 1758), and a sphaerodactylid *Euleptes europaea* (Gené, 1839) (Bauer et al. 2008). *Euleptes europaea*, the focus of the present study, commonly known as the European leaf-toed gecko, the single living species of the genus *Euleptes*, which was recently resurrected from synonymy with of *Phyllodactylus* (Bauer et al. 1997). Moreover, not long ago, this monotypic genus was considered *incertae sedis*, along with few other Afro-Eurasian genera of the same clade (*Pristurus* Rüppell, 1835, *Teratoscincus* Strauch, 1863, *Quedenfeldtia* Boettger, 1883) plus neotropical *Aristelliger* Cope, 1861, because they all fall into an unresolved polytomy (Gamble et al. 2008a, 2008b). The most up to date phylogeny of Gamble et al. (2011), however, places this monotypic genus into Sphaerodactylidae, once again raised to a rank of a family, which is a sister clade to Gekkonidae and Phyllodactylidae and embraces a large range of species from both Old and New World.

In Europe, *Euleptes* Fitzinger, 1843 is described from at least the early Miocene; the single modern species, *Euleptes europaea*, is a relic endemic of the western Mediterranean region which survived during isolation on the Corso-Sardinian microplate (Müller 2001). In contrast with the other three species widespread on the Italian territory, the current geographic range of *Euleptes europaea* is restricted to Sardinia, Corsica, small mainland and insular areas of Liguria and Tuscany, including the isles of Elba, Gorgona, Capraia, Pianosa, Montecristo, Giglio, and Giannutri, and also to small offshore islands of southern France, Sardinia, and Corsica (Sindaco et al. 2006), as well as to three islands of the Tunisian coast (Delaugerre et al. 2011). This peculiar, largely insular, distribution indicates a relatively recent contraction of its range (Arnold and Ovenden 2002).

*Euleptes europaea* remains the only gecko of the Italian fauna, which has not been characterized cytogenetically. It is not surprising, since of approximately 1,000 species of Geckonids, in the broad sense, karyotypes have been described for less than 10% of them (Olmo and Signorino 2005, Trifonov et al. 2011). Cytogenetic data are very scarce in Sphaerodactylidae, as well: only 3% of approximately 196 species have been karyotyped (Ezaz et al. 2009). Accordingly, we carried out cytogenetic analyses of *Euleptes europaea* performing a karyological description of individuals from Sardinia, supplemented by physical mapping of telomeric repeats. Molecular cytogenetic investigations on reptiles are largely lacking, but they may be beneficial to solving taxonomic problems and phylogenetic uncertainties and to comprehending evolutionary matters, such as the mechanisms of chromosome evolution and emergence of neo-sex chromosomes, especially in geckos, which are characterized by different sex-determination systems even among closely related taxa (Gamble 2010, Kawai et al. 2009).

## Materials and methods

We analyzed a limited sample - one male, one female, and one juvenile - from a population of the island of Santa Maria near the north coast of Sardinia. The animals were handled according to the European Code of Practice for the housing and care of animals used in scientific procedures (Council of Europe 1986). Analyzed specimens (voucher numbers: EUL1 male, EUL2 juvenile, EUL3 female) are preserved in 70% ethanol and are housed in the herpetological collection of the Dipartimento di Biologia e Biotecnologie “Charles Darwin” Università di Roma “La Sapienza” (CEAC).

Metaphase plates were prepared from bone marrow, intestinal, and testicular cells using standard air-drying method after injection of 1:1000 solution Vinblastine Sulphate, Velbe® (Lilly) as antimitotic solution. The slides were colored with 5% Giemsa solution. For each individual, about 20 metaphase plates were studied and photographed. The telomeric probe was commercially synthesized as two oligonucleotides (GGGTTA)_7_ and (TAACCC)_7_ both end-labeled with Cy3 (Bio-Fab Research). The oligonucleotides were dissolved (2 ng/µL) in a hybridization mix made up of 5% Dextran sulphate, 2XSSC, and 5 µg/µL sonicated salmon DNA. For FISH, standard procedures for the hybridization of repetitive sequences (Lichter et al. 1992) were carried out, followed by high-stringency post-hybridization washes at 42°C. As a routine, chromosomes were counterstained with DAPI (4’, 6-diamidin-2-fenilindolo, 1µg/mL) and propidium iodide (0.5 µg/mL). Ten metaphases per individual were analyzed under Zeiss AxioPhot epifluorescence microscope. The photographs were acquired with a SenSys 1400 CCD camera (Photometrics®). Images were processed using IP-lab software (Scanalytics®) and Adobe® Photoshop® CS3.

## Results and discussion

**Figure 1.**
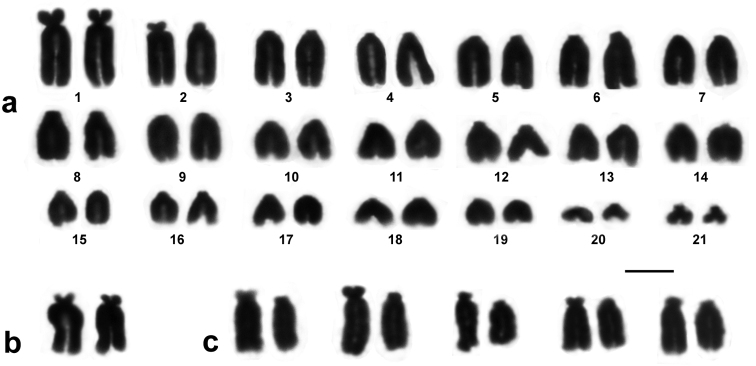
Chromosome complement of *Euleptes europaea* from Sardinia. **a** 2n = 42 male karyotype **b** homomorphic chromosomes 2 (female); **c** – examples of heteromorphic chromosomes №2 (male). Bar = 5 µm.

The karyotype of *Euleptes europaea* is composed of 21 chromosome pairs gradually decreasing in size (Fig. 1a). There is no pronounced subdivision of the chromosome complement into macro- and microchromosomes; 17 chromosome pairs may be considered telocentric chromosomes: tiny short arms, visible in some of more elongated chromosomes, are not taken into account for the fundamental number. The minute chromosomes № 20 and № 21 are telocentric, while the smallest pair of the karyotype is definitely biarmed. The largest chromosomes of the complement (pairs № 1 and № 2) are also biarmed, precisely, subtelocentric. However, both homologues of the chromosomes № 2 had short, similar in size true arms only in the female individual (Fig. 1b). In the male, one of the homologues of chromosomes № 2 showed somewhat greater overall compactness and smaller or more contracted short arms in most metaphases after conventional Giemsa staining (Fig. 1c). The degree of this heteromorphism was relevant enough to be worth noting: the average centromeric index of the two homologues of this pair was 14.7% and 8.3%.

**Figure 2.**
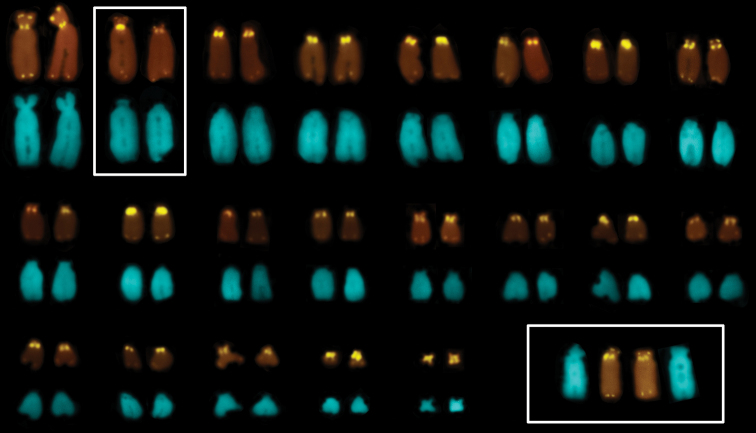
A karyotype of *Euleptes europaea* after FISH with a telomeric probe (upper array) and DAPI-staining (lower array); slightly heteromorphic chromosomes № 2 are framed; the same chromosome pair of a female is shown in the insert below.

FISH with a telomeric probe detected all ordinary telomeric sites of the chromosomes. The present results are in accordance with previously obtained data in *Gonatodes taniae* Roze, 1963, the only other sphaerodactylid species, in which chromosomal distribution of telomeric sequences has been studied so far (Schmid et al. 1994). Also, amplification of the telomeric signals characterized most of telocentric chromosomes in centromeric regions (Fig. 2). This pattern, together with DAPI counterstaining, allowed to better classify chromosomes and arrange homologues in pairs. In the obtained karyotype, conspicuous interstitial pericentromeric signals are clearly separated from minor regular telomeres in the biarmed chromosomes and in several chromosomes with tiny short arms (e.g., № 8 and № 13 in Fig. 2). Furthermore, in all chromosome pairs, interstitial telomeric sites (ITS) are virtually of the same intensity and size in both homologues, whereas the two homologues of the chromosomes №2 of the male differ for the intensity of interstitial signals.

The ITS sites at centromeres have been described in many different taxonomic groups (Meyne et al. 1990). In some lineages, they were shown to result from retaining the ancestor telomeres after, for example, Robertsonian or tandem fusion/fission (Ventura et al. 2006) or more complex (Fagundes et al. 1997) rearrangements. On the other hand, telomere-like sequences are often present in chromosomes as a component of the satellite DNA (Garrido-Ramos et al. 1998). In many species, centromeric regions of chromosomes contain substantial amounts of telomeric repeats, which often constitute a major component of heterochromatin and is supposed to play an important role in evolutionary chromosomal rearrangements (Slijepcevic et al. 1997, Ruiz-Herrera et al. 2008).

In summary, the karyotype of *Euleptes europaea* looks quite unusual if compared with other records available in the family Sphaerodactylidae, and the chromosome number is the highest among all species of the family presently studied. Since the phylogenetic position of *Euleptes* within Sphaerodactylidae is uncertain, we provide a comparative analysis of all-encompassing data. The genus *Euleptes* falls in a poorly supported assemblage of genera without clear relationships with each other, which includes the following species-poor Afro-Asian genera: *Pristurus* Rüppell, 1835, endemic to Middle East and Arabia, the Asiatic *Teratoscincus* Strauch, 1863, and the Moroccan *Quedenfeldtia* Boettger, 1883, as well as the neotropical species-rich *Aristelliger* Cope, 1861 (Gamble et al. 2011). Among these taxa, *Teratoscincus scincus* (Schlegel, 1858) from several Chinese populations (Zheng et al. 1998) and its two subspecies (*Teratoscincus scincus scincus* and *Teratoscincus scincus rustamowi*) from the Central Asia (Kazakhstan, Tadjikistan, and Turkmenia) (Manilo 1993, Manilo and Pisanets 1984), as well as *Teratoscincus przewalskii* Strauch, 1887 (Zheng et al. 1998), all have a 2n = 36 karyotype. The results of different authors are in accordance with each other in presenting a karyotype formula of 2n = 36, with 24 macrochromosomes (6 biarmed and 18 telocentric) and 12 microchromosomes, except for a pioneer result of De Smet (1981), who reported a karyotype of 2n = 34 all acrocentric chromosomes in *Teratoscincus scincus* (Schlegel, 1858). According to Branch (1980), *Pristurus carteri* (Gray, 1863) have similar, 2n = 34 all-acrocentric karyotype.

The family Sphaerodactylidae includes also one well supported major clade, which comprises five genera of the neotropical sphaerodactylid lizards (*Coleodactylus* Parker, 1926; *Gonatodes* Fitzinger, 1843; *Lepidoblepharis* Peracca, 1897; *Pseudogonatodes* Ruthven, 1915, and *Sphaerodactylus* Wagler, 1830) (see dos Santos et al. 2003). The highest diploid number of chromosomes within this cluster is 32. Thus, three species of *Gonatodes* (*Gonatodes humeralis* (Guichenot, 1855), *Gonatodes hasemani* Griffin, 1917, and *Gonatodes vittatus* (Lichtenstein, 1856)) and *Coleodactylus amazonicus* (Andersson, 1918) show 2n = 32, all telocentric karyotypes (McBee et al. 1984, 1987, Rada De Martinez 1980, dos Santos et al. 2003), but some species of *Gonatodes* have lower diploid number (2n = 22 and 26 in *Gonatodes ceciliae* Donoso-Barros, 1966) (McBee et al. 1987) or exceptionally low one (2n = 16 in *Gonatodes taniae* Roze, 1963), which is thought to be due to a series of centric fusions from an acro(telo)centric ancestral karyotype (Schmid et al. 1994). Based on its prevalence among the neotropical sphaerodactylid geckos, the 2n = 32 all-acrocentric karyotype was proposed as ancestral, while centric fusion was assumed as the main mechanism of chromosome evolution in this latter grouping (Schmid et al. 1994). On the other hand, once, considering the family Gekkonidae, then inclusive of sphaerodactylid lizards, King (1977) suggested as ancestral a 2n = 38 karyotype with exclusively acrocentric chromosomes. Taking in account the present evidence of the 2n = 42 karyotype of *Euleptes europaea* with mainly telo(acro)centric chromosomes, we must agree with dos Santos et al. (2003) that it is still premature to speculate on the ancestral karyotype for Sphaerodactylidae.

Another outcome of the present study is a possible male chromosome heteromorphism in *Euleptes europaea*. However, provided the extremely limited sample presently examined, chromosome polymorphism unrelated to sex is possible, as well. If the present data in *Euleptes europaea* actually reflect the XX/XY sex determination system, which is still to be corroborated, it would be indicative of rather new or undifferentiated sex chromosomes. The available cytogenetic data on sex chromosomes in Sauria are rare, but give an idea of how different may be the morphology and composition of sex chromosomes in different species with male (XX/XY) or female (ZZ/ZW) heterogamety (Ezaz et al. 2009). Among few karyotyped geckos of Spherodactylidae, no female heterogamety has been found yet, while male heterogamety has been reported in only one species – the Venezuelan *Gonatodes ceciliae* Donoso-Barros, 1966 (McBee et al. 1987). However, in this species, a large metacentric X and a small acrocentric Y chromosome are remarkably heteromorphic. Finally, a genetic sex determination system may be hypothesized in a lizard species, which inhabits particular environment, as very small islets and isolated rocks. Such environment possibly will not provide consistent temperature ranges, which are necessary to assure a balanced sex ratio within population (Pen et al. 2010). In fact, *Tarentola mauritanica*, which is known to have environmental sex determination, has not been found on islets so small as the ones, where the *Euleptes europaea* is often observed (Delaugerre et al. 2011).

The main conclusions of the present analysis are: 1) the diploid chromosome number in Sphaerodactylidae may reach 2n = 42, the uppermost value so far observed in the family, as well as one of the highest diploid numbers among all Gekkotan lizards (acknowledged maximum is 2n = 46 in Thailand house gecko, *Cosymbotus platyurus* (Schneider, 1792) (classified also as *Hemidactylus platyurus* (Schneider, 1792)) according to Olmo and Signorino (2005), as well as in *Hemidactylus bowringi* (Gray, 1845) according to Nakamura (1932) and Ota (unpublished) (in Ota et al. 1989)), whereas even higher numbers of chromosomes characterize some unisexual triploid lineages, e.g., the parthenogentic gecko *Hemidactylus stejnegeri* Ota et Hikida, 1989 (3n = 56) or *Hemidactylus vietnamensis* Darevsky et al., 1984 (3n = 60) or *Hemidactylus garnotii* Duméril & Bibron, 1836 (3n = 70) (see Ota et al. 1989); 2) centromeric regions of all chromosomes of *Euleptes europaea* are rich in telomeric repeats, which may play an active role in the karyotype evolution of the lineage; 3) on the base of likely heteromorphism of chromosome pair № 2, a male heterogamety may be tentatively hypothesized in *Euleptes europaea*.
